# Pain modulation profiles in temporomandibular disorders with migraine and fibromyalgia

**DOI:** 10.22514/jofph.2026.012

**Published:** 2026-01-12

**Authors:** Pankaew Yakkaphan, Tara Renton

**Affiliations:** ^1^Faculty of Dentistry, Prince of Songkla University, 90110 Songkhla, Thailand; ^2^Kings College London, SE1 9RT London, UK

**Keywords:** Temporomandibular disorders, Conditioned pain modulation, Pain modulation profiles, Pain comorbidities, Migraine, Fibromyalgia

## Abstract

**Background**: Temporomandibular disorders (TMD) frequently occur with other pain conditions, and existing research has yielded mixed results regarding the presence or absence of endogenous pain modulation (EPM). This study aimed to investigate EPM in TMD patients with comorbid migraine and fibromyalgia (FM) in the non-trigeminal innervated area. **Methods**: Conditioned pain modulation (CPM) and temporal summation (TS) were assessed in healthy controls (n = 30), TMD without comorbidity (n = 30), migraine (n = 30), TMD with migraine (n = 30), and TMD with migraine + FM (n = 19). Based on the TS and CPM responses, participants were categorized into pain modulation profiles (PMP, I–IV). **Results**: In serial stimulation, patients with migraine, TMD + migraine, and TMDs + migraine + FM showed significantly reduced pain inhibition compared with controls (*p* = 0.003, *p * < 0.001, and *p* = 0.001, respectively), while TMD patients without comorbidity exhibited intact modulation. Increasing comorbidities were also linked to weaker CPM (single stimulation: *R* = −0.312, *p * < 0.001; serial stimulation: *R* = −0.344, *p* < 0.001). The PMP categorization demonstrated distinctions among the study population, although further subgroup analysis proved challenging. **Conclusions**: TMD patients without comorbid pain exhibited intact EPM in non-painful areas. However, when these individuals experience comorbid pain conditions, their ability to modulate pain may be compromised due to the pain amplification and central sensitization associated with multiple comorbid pain conditions.

## 1. Introduction

The nociceptive and pain-modulatory pathways enable the brain to regulate pain sensations through both inhibitory and excitatory processes [1]. One of the outputs of the central nervous system (CNS) to modulate pain is the endogenous pain modulation (EPM). Abnormal EPM, either increased pain facilitation and/or impaired pain inhibition, might be associated with the genesis of several chronic pain mechanisms [2, 3]. EPM can be assessed by using psychophysical methods, such as temporal summation of pain (TSP) and conditioned pain modulation (CPM) [4]. TSP evaluates facilitatory modulation through the changes in pain perception caused by a series of repeated noxious stimuli [4, 5]. CPM is a paradigm used to assess the pain inhibition pathway based on the “pain inhibits pain” theory [6].

Temporomandibular disorders (TMD) are a collective term for a heterogeneous condition of musculoskeletal disorders involving pain and/or functional limitations in the masticatory muscles, temporomandibular joints (TMJ), and associated structures in the orofacial region [7, 8]. Multiple studies have assessed pain facilitation and modulation in TMD patients in both trigeminal and non-trigeminal areas. Some studies reported higher sensitivity to noxious stimuli in TMD patients compared with healthy controls [9, 10]. In addition, certain studies reported lower pain thresholds [11, 12] and increased TSP in TMD patients when tested outside the trigeminal nerve region [13]. In contrast, another study found no significant difference in pain thresholds outside the painful area [14]. Regarding CPM responses, TMD cases produced impaired CPM at extra-segmental sites compared with controls [15, 16]. In other studies, however, no significant change in the CPM effect was found at pain-free sites between those with and without TMD [17, 18].

Migraine is a head pain frequently characterised by unilateral throbbing pain and a variety of neurological and autonomic symptoms, including hypersensitivity to light, sound, smell, nausea, cognitive, emotional, and motor disturbances [19]. Studies regarding the investigation of CPM efficiency in migraine patients have reported mixed results. A preliminary study showed disrupted descending pain modulation in both episodic and chronic migraine [20], while other CPM studies indicated that migraine patients typically show only mild or absent inhibitory responses, comparable to those in controls [21].

Fibromyalgia is characterised by widespread musculoskeletal pain, tenderness, and heightened pain sensitivity, often accompanied by other symptoms, such as fatigue, sleep disturbances, and cognitive issues [22]. CPM responses in individuals with fibromyalgia have been somewhat inconsistent. Some studies, on one hand, have suggested that individuals with fibromyalgia exhibit impaired CPM responses [23]. On the other hand, other research has reported normal [24] or even enhanced CPM responses in fibromyalgia patients [25].

TMD is associated with several comorbidities, particularly migraine and fibromyalgia. The three pain conditions have been associated with impaired EPM and they occasionally coexist in some patients. To date, no studies have measured the EPM system in TMD patients with comorbid migraine and fibromyalgia. Considering previous findings, we hypothesised that TMD patients with comorbidities may exhibit greater EPM impairment in the non-trigeminal innervated area compared with TMD patients without comorbidities. Therefore, this study aimed to investigate EPM in TMD patients with comorbid migraine and fibromyalgia (FM) in a non-trigeminally innervated area.

## 2. Materials & methods

### 2.1 Sample size

A previous meta-analysis, which encompassed 9 studies exploring pain modulation in chronic orofacial pain, these studies comprised 14 to 58 samples within their respective groups, with an average sample size of 20 to 30 participants. The findings from these studies demonstrated compromised pain modulation in patients compared with control groups [13]. A power analysis was conducted using G*Power version 3.1.9.4 (Heinrich-Heine-Universität Düsseldorf, Düsseldorf, NRW, Germany) to ascertain the minimum sample size essential for testing the study hypothesis. The results indicated that to achieve 80% power for detecting a medium effect, at a significance level of α = 0.05, a total sample size of N = 269 was necessary, equating to 54 individuals per group. Hence, considering insights from previous studies and the power analysis calculation, it was recommended to conduct the study with a sample size ranging from 15 to 54 participants per group. Ultimately, the study succeeded in enrolling a total of 135 participants, which were 30 participants in four groups and 15 participants in one group.

### 2.2 Participants

Participants were recruited between May 2021 and December 2023 from Orofacial Pain Clinic, King’s College Dental Institute and Headache Clinic at St Thomas’ Hospital. Ethical approval for the study was obtained from the Health Research Authority (Approval No: 21/WS/0050). Informed consent was mandatory prior to participation.

#### 2.2.1 Inclusion criteria

Participants were recruited into five groups based on history and clinical examination:

● TMD: chronic myogenous painful TMD patients. TMD diagnosis was based on the 1st edition of International Classification of Orofacial Pain, 1st edition (ICOP) for primary myofascial orofacial pain [26] and TMD pain presented for more than 3 months.

● Migraine (MG): chronic migraine patients. Chronic migraine diagnosis followed the 3rd edition of the International Classification of Headache Disorders (ICHD-3) [19].

● (TMD + MG): chronic myogenous painful TMD patients with chronic migraine.

● (TMD + MG + FM): chronic myogenous painful TMD pain patients with comorbid migraine and fibromyalgia. Fibromyalgia diagnosis aligned with the American College of Rheumatology (ACR) criteria [22].

● Healthy participants.

The study was inclusive of individuals aged between 18 and 50 years complying with specific inclusion and exclusion criteria. This selection aimed to mitigate the influence of confounding factors related to the experimental protocol [3] and the participant characteristics [27, 28, 29] in the context of the CPM paradigm.

#### 2.2.2 Exclusion criteria

● Presence of systemic comorbidities and psychological disorders, for example, fibromyalgia, systemic myofascial pain and chronic fatigue syndrome, chronic headaches, migraines, heart arrhythmias, endometriosis, interstitial cystitis, irritable bowel syndrome, lower back pain, autoimmune diseases, and sleep disorders or diabetes mellitus.

● Diagnosis of medication overuse for headache, and use nonsteroidal anti-inflammatory drugs (NSAIDs) or paracetamol within 12 hours before the experiment.

● Smoking more than five cigarettes daily.

● Consuming over 6 cups of caffeinated drinks daily.

● Indications of substance abuse.

● Alcohol consumption within 24 hours before the experiment.

● Irregular menstrual cycles in the case of female participants.

### 2.3 Study method

The study was divided into three parts, as follows:

Part 1: Mechanical detection threshold (MDT) and pressure pain threshold (PPT).

Part 2: Mechanical Temporal Summation (MTS) and decay of after-sensations.

Part 3: Conditioned Pain Modulation Protocol (CPM).

#### 2.3.1 Part 1: mechanical detection threshold (MDT) and pressure pain threshold (PPT)

To measure mechanical detection threshold (MDT), von Frey Filaments (Semmes-Weinstein) were administered on both the painful site and the hands. The procedure began with the application of a filament with an initial force of 0.008 grammes (0.08 mN). With eyes closed, participants were asked to indicate when they first sensed the touch. The MDT with von Frey Filaments is a standard tool for assessing tactile and mechanical pain thresholds. High test-retest reliability has been reported (intraclass correlation coefficients (ICC) 0.76–0.98), though some studies note only moderate reproducibility (Kappa <0.6), influenced by examiner training, site, and protocol [30, 31].

Subsequently, the pressure pain threshold (PPT) was assessed by a pressure algometer (Pressure algometer, Wagner Pain Test™, Wagner Instruments, Greenwich, CT, USA) at the same locations. The PPT by algometry showed high intra- and inter-rater reliability, with excellent concurrent validity between digital and analogue devices (ICC 0.82–0.99) [32]. Pressure was manually increased at a rate of 30 kPa per second until participants reported feeling the first sensation of pain. Each stimulation was repeated three times and then the average force was calculated.

#### 2.3.2 Part 2: mechanical temporal summation (MTS) and decay of after-sensations

The MTS was performed to evaluate pain facilitation (Fig. 1). The test was conducted according to the Deutscher Forschungsverbund Neuropathischer Schmerz (DFNS) standardised protocol [33] by using weighted pinprick stimulators (Pinprick Stimulator, MRC Systems GmbH, Heidelberg, BW, Germany). Employing the recommended force of 256 mN for the hand [34], a pinprick stimulus was applied over the volar part of the forearm of the dominant hand. The stimulator was positioned vertically, perpendicular to the testing surface, and a single pinprick stimulus was administered. This stimulus was repeated three times, each with a 10-second interval. After each pinprick test, patients were asked to rate the painful sensation on a numerical pain scale (NPS) of 0 to 100, where 0 represents no pain and 100 signifies the maximum imaginable pain. After a single pinprick test, this was followed by a train of 10 successive stimuli, 3 cycles of the same force and repeated at a 1/s rate (1 Hertz). The repetition of stimuli was concentrated within a confined area of 1 cm^2^. After every 10 stimuli, patients were asked to rate the degree of painful sensation on a numerical pain scale. Subsequently, the wind-up ratio (WUR) was computed by dividing the mean rating of the three series of 10 stimuli by the mean rating of the three individual stimuli. The MTS has demonstrated good to excellent test-retest reliability in both healthy and clinical populations. For example, ICC values range from 0.80–0.91 in healthy subjects (hand/back sites) using 2–3 repeated measurements [35], 0.73–0.89 in low back pain patients [36], and 0.63–0.86 in shoulder pain cohorts [37].

**Fig. 1.** Overview of mechanical temporal summation and conditioned pain modulation protocol.

#### 2.3.3 Part 3: conditioned pain modulation protocol (CPM)

Each participant underwent the CPM paradigm (Fig. 1); the pinprick stimulator used in the MTS test served as a test stimulus in the CPM test, while cold water served as the conditioning stimulus (CS). This specific cold water conditioning stimulus aligned with the recommendations from the CPM guidelines [38]. The selection of a 10 °C intensity followed a precedent set by a previous protocol and has been validated as effectively inducing a CPM effect through corroborating studies [39, 40]. Cold water was prepared within an insulated ice bucket, and its temperature was measured with a digital thermometer. Participants were requested to immerse their nondominant hand in cold water at wrist level. Blood pressure and pulse rate were measured at the 20-second mark. At the 30-second point during the immersion, the MTS procedure was applied using the same protocol as previously described, but targeting a different area on the same hand. CPM magnitude was calculated as the difference of pain intensity report during the hand immersion in cold water and pain intensity report without hand immersion from the MTS test (CPM = MTS with conditioning pain score − MTS without conditioning pain score). A negative CPM value indicated a decrease in pain response to the TS when a simultaneous CS is applied. The % CPM effect was calculated by determining the percentage reduction in pain perception caused by the conditioning stimulus compared to the baseline stimulus. The formula used was: % CPM effect = ((baseline pain − conditioning stimulus pain)/baseline pain) × 100. A higher % CPM effect indicated a stronger pain modulation response, meaning the body is better at inhibiting pain perception in response to the conditioning stimulus. For the validity and reliability of CPM, a meta-analysis reported good intra-session reliability (ICC 0.64–0.77 in healthy subjects; ICC 0.77 in patients), but only fair inter-session reliability (ICC 0.44–0.59), depending on test and conditioning stimuli [41].

To determine an efficient CPM, two approaches were used to determine the % CPM threshold. Firstly, a review conducted by Pud *et al*. [42] revealed that the expected reduction in pain sensitivity corresponding to the Diffuse Noxious Inhibitory Control (DNIC) effect, averaged approximately 29%. This finding implied that when participants experienced one pain-inducing stimulus, they subsequently experienced another pain-inducing stimulus to a lesser degree. As a result, efficient conditioned pain modulation was characterised as the ability of individuals to suppress at least 29% of pain and this cutoff was applied in a later study [43]. Secondly, the method proposed by Locke *et al.* [44] was adopted and further elaborated in the section on pain modulation profile (PMP) classification.

### 2.4 Pain modulation profile (PMP) classification

The classification of participants into different categories of PMP was achieved by applying the cut-off thresholds of WUR and CPM [45]. PMP refers to different ways in which an individual’s body responds to and regulates pain signals [46]. In the context of WUR, the cut-off values were determined by adjusting the method described by Vaegter and Graven-Nielsen [45]. This adjustment process involved the inclusion of normative temporal summation data obtained from a cohort of 110 healthy women. Individual WUR values that exceeded the upper limit (95% confidence interval) of the normative WUR data for the hand (WUR >2.57) were identified as elevated. For the establishment of CPM cut-off criteria, a methodology described by Locke *et al*. [44] was implemented. This approach integrated temporal summation (TS) data collected across three experimental cycles, alongside the CPM data. A significant CPM effect was recognised when the percentage increase in TS from the baseline exceeded the inherent measurement error. The calculation of the measurement error involved a series of steps. Firstly, the intraclass correlation coefficient (ICC) model 3.3 for mean TS (both single and serial stimulation) within each group (5 groups) from the three experimental cycles. Then, computed the standard error of measurement (SEM) for each visit, using the formula: SEM = SD(TS) × square root of (1 − ICC), and the sum of the SEM with the mean TS for each stimulation. Next, we converted this value into a percentage change relative to the mean TS. Finally, we averaged these three TS + SEM relative change percentage values. Any CPM value above the inherent measurement error indicated a normal or greater effect. By applying those WUR and CPM effect cut-off values, we therefore classified participants into four different PMP categorizations: PMP I: Double pro-nociception (increased TSP/impaired CPM), PMP II: Inhibitory pro-nociception (normal TSP/impaired CPM), PMP III: Facilitatory pro-nociception (increased TSP/normal CPM) and PMP IV: Antinociception (normal TSP/normal CPM).

### 2.5 Data analysis

All manual data obtained was entered into a secure electronic database. Categorical variables were analysed using the chi-square test. Continuous outcomes were assessed for the normality, and reported as mean ± standard deviation (SD) with 95% confidence interval. Mean differences among 5 groups were compared using analysis of variance (ANOVA) if data was normally distributed and Kruskal-Wallis H test for non-normally distributed data. The significance level was set at *p * < 0.05. Additionally, the *post hoc* test was conducted when the initial analysis demonstrated statistical significance.

## 3. Result

### 3.1 Participants

Of the 152 participants, 13 were excluded for specific reasons: 4 had taken NSAIDs within 5 hours before the study, 4 had consumed alcohol within the previous 12 hours, 3 had smoked cigarettes before the experiment, and 2 were unable to tolerate the water temperature. Therefore, the final number of participants included in the study was 139. The clinical characteristics of participants are shown in Table 1. Among them, 99 were female and 40 were male, with a mean age of 42.13 years, SD 10.81. Participants had a mean BMI of 23.15, SD 3.58. Most participants (96%) were right-handed. The CPM protocol was performed predominantly between 13:00 and 17:00. The average temperature of the cold water used in the test was 9.96 °C with an SD of 0.42 °C. No significant differences were observed across the various characteristics of the participants. When examining cases, the TMD + MG + FM group gave the highest mean clinical pain score of 60 out of 100.

**Table 1.** General characteristics of study participants.

Characteristics	Total	Control (n = 30)	TMD (n = 30)	MG (n = 30)	TMD + MG (n = 30)	TMD + MG + FM (n = 19)	*p*-value
Age (yr), mean ± SD	42.13 ± 10.81	42.77 ± 8.46	39.73 ± 11.61	42.10 ± 12.91	42.17 ± 11.88	44.89 ± 7.05	0.604
Female:Male (n)	99:40	18:12	20:10	22:8	24:6	15:4	0.421
BMI, mean ± SD	23.15 ± 3.58	22.22 ± 4.85	23.01 ± 2.78	23.93 ± 3.71	22.63 ± 3.15	24.42 ± 2.29	0.163
Handedness	Left-handed = 11 Right-handed = 128	Left-handed = 3 Right-handed = 27	Left-handed = 3 Right-handed = 27	Left-handed = 3 Right-handed = 27	Left-handed = 1 Right-handed = 29	Left-handed = 0 Right-handed = 19	0.552
Pain score before testing (0–100), mean ± SD	N/A	0	49.33 ± 15.07	50.50 ± 14.64	58.67 ± 13.58	60.00 ± 13.74	N/A
Thermal probe temperature (°C), mean ± SD	9.96 ± 0.42	10.04 ± 0.48	10.01 ± 0.46	9.87 ± 0.44	9.88 ± 0.35	10.04 ± 0.32	0.340
Time of testing day	Morning = 58 Afternoon = 81	Morning = 17 Afternoon = 13	Morning = 12 Afternoon = 18	Morning = 12 Afternoon = 18	Morning = 9 Afternoon = 21	Morning = 8 Afternoon = 11	0.340

*TMD: TMD patients; TMD + MG: TMD patients with chronic migraine; MG: Chronic migraine patients; TMD + MG + FM: TMD patients with comorbid migraine and fibromyalgia. SD: Standard Deviation; TMD: Temporomandibular disorders; MG: migraine; FM: fibromyalgia; N/A: Not Applicable.*

MDT and PPT values and differences were documented in Fig. 2a,b. Within the participant pool, individuals with migraine (MG) had the lowest mean threshold of 0.45 grammes on the face, while the TMD + MG + FM group had the lowest mean threshold of 0.68 grammes on the hand. In contrast, the remaining groups exhibited relatively similar mean MDT values for both facial and hand areas. Significant differences of MDT were detected when accessing the painful region, differentiating between the MG group from the healthy controls, the TMD, as well as the TMD + MG groups. In terms of the PPT test, the MG group consistently reported the lowest threshold on the face (1.35 kPa), whereas the control group showcased the highest PPT (1.59 kPa). Nevertheless, distinctions were also apparent in testing on the face between when comparing control group with the MG, TMD + MG, and TMD + MG + FM groups. Interestingly, although the TMD + MG + FM group registered the lowest PPT on the hand, no significant differences were observed across all five groups.

### 3.2 Temporal summation (TS) and wind-up ratio (WUR)

The TMD + MG + FM group had the most elevated pain scores of 21 and 43 out of 100 in the single and serial tests, respectively. In contrast, the healthy control group reported the lowest pain scores in both assessments, 15 and 32 out of 100, respectively (Table 2). No significant differences were found between the participants when the TS test was examined with a single stimulus. However, with serial stimulation, there were notable differences between the groups. The mean WUR from the TS tests was presented in Table 2 and Fig. 2c, but the result showed no significant differences across the five study groups.

**Table 2.** Pain rating scores and outcomes during temporal summation and conditioned pain modulation protocol.

Parameters		Control (n = 30)	TMD (n = 30)	MG (n = 30)	TMD + MG (n = 30)	TMD + MG + FM (n = 19)	*p*-value
Single stimulation NRS (0–100)							
	Temporal summation	15.61 ± 6.45	20.56 ± 7.13	16.70 ± 7.60	19.39 ± 8.54	21.05 ± 7.56	0.060
	Conditioning stimulus	7.87 ± 6.24	16.00 ± 8.03	13.30 ± 6.50	15.17 ± 8.65	17.63 ± 6.74	<0.001*
	With-in group (*p*-value)	<0.001*	<0.001*	0.015*	0.005*	0.055	
Serial stimulation NRS (0–100)							
	Temporal summation	32.46 ± 12.60	38.22 ± 7.30	36.20 ± 11.66	37.67 ± 9.16	42.98 ± 7.85	0.003*
	Conditioning stimulus	21.10 ± 12.65	30.72 ± 11.09	31.08 ± 11.04	33.44 ± 9.16	39.65 ± 12.43	<0.001*
	With-in group (*p*-value)	<0.001*	<0.001*	0.006*	0.011*	0.102	
CPM magnitude (Mean ± SD)							
	Single stimulation	−7.74 ± 6.47	−4.55 ± 5.44	−3.40 ± 7.16	−4.22 ± 7.65	−3.95 ± 7.11	0.135
	Serial stimulation	−11.36 ± 9.42	−7.50 ± 7.87	−5.12 ± 9.37	−4.22 ± 8.48	−3.95 ± 7.35	0.012*
Wind Up Ratio (WUR)		2.22 ± 0.87	2.04 ± 0.67	2.40 ± 0.76	2.30 ± 1.10	2.27 ± 0.83	0.371
CPM effect change (% CPM)							
	Single stimulation	47.71 ± 40.21	22.05 ± 27.78	9.62 ± 55.50	17.39 ± 48.81	11.10 ± 35.32	0.011*
	Serial stimulation	33.01 ± 31.44	21.37 ± 23.01	12.15 ± 26.79	8.33 ± 26.54	9.08 ± 22.44	0.001*
Frequency of participants with efficient CPM (inhibit ≥29%)							
	Single stimulation	21 (70.00%)	13 (43.33%)	13 (43.33%)	13 (43.33%)	7 (36.84%)	0.110
	Serial stimulation	18 (60.00%)	13 (43.33%)	11 (36.67%)	7 (23.33%)	4 (21.05%)	0.016*

*: Statistical difference (*p * < 0.05); TMD: TMD patients; MG: Chronic migraine patients; TMD + MG: TMD patients with chronic migraine; TMD + MG + FM: TMD patients with comorbid migraine and fibromyalgia. *NRS: Numeric Rating Scale; SD: Standard Deviation; CPM: conditioned pain modulation; TMD: Temporomandibular disorders; MG: migraine; FM: fibromyalgia.*

### 3.3 Conditioned pain modulation (CPM) testing

The pain scores obtained from the CPM experiment were detailed in both Table 2 and Fig. 2d. The control group reported the highest pain scores, and these scores differed significantly from the other four groups for both single and serial stimulation, 8 and 21 out of 100, respectively. In the context of within-group analysis (Table 2), the presence of pain inhibition (where the pain score during CPM deviated from the baseline pain score in MTS) was evident in all groups for both types of stimulation, except for TMD + MG + FM in both stimulations.

The CPM magnitude values for both the single and serial tests were presented in Table 2 and Fig. 2e. No noticeable distinction in CPM magnitude emerged for the single stimuli. However, in the context of serial stimulation, the control group showed the lowest CPM magnitude (−11), which was significantly different from the MG, TMD + MG and TMD + MG + FM groups. Furthermore, apart from the healthy participants, the TMD group demonstrated the second lowest CPM magnitude (−8) during serial stimulation, followed by the MG (−5), TMD + MG (−4) and TMD + MG + FM (−4) groups.

### 3.4 Percentage of CPM effect change

The pain score acquired from the MTS and the CPM were used to calculate the percentage change in CPM effect. The CPM effect was reversed from CPM magnitude which meant that higher CPM effect (more positive percentage) indicated higher pain inhibition ability, while lower CPM percentage indicated lower pain inhibition ability. The mean percentage change within each group was reported in Table 2 and the significant differences among groups were shown in Fig. 2f. In the single stimulation, CPM effect in healthy individuals was higher and different from other groups of patients. For the serial stimulation, all the pain patients, except for TMD patients, exhibited lower CPM effect than controls and the difference was also observed between TMD and TMD + MG groups. The frequency of participants who had efficient CPM (the ability of individuals to inhibit at least 29% of pain) were presented in Table 2 and Fig. 3. It illustrated that the largest proportion of participants with effective CPM responses were found among the healthy samples, accounting for 70% and 60% for the single and serial tests, respectively. In contrast, the lowest proportion of effective CPM responders was observed in the TMD + MG + FM group, with rates of 36% and 21% for the single and serial tests, respectively.

**Fig. 2.** Comparison of (a) mechanical detection threshold (MDT), (b) pressure pain threshold (PPT) among participants, (c) pain score during mechanical temporal summation (TS), (d) pain score during conditioned pain modulation (CPM), (e) conditioned pain modulation magnitude, (f) percentage of CPM effect change. Controls (black), TMD Patients (orange), Migraine Patients (green), TMD Patients with Comorbid Migraine (blue), TMD Patients with Comorbid Migraine and Fibromyalgia (grey). SD: standard deviation; TMD: temporomandibular disorder; MG: migraine; FM: fibromyalgia.

**Fig. 3.** Percentage of participants with an effective conditioned pain modulation (CPM) response, a minimum of 29% pain inhibition. TMD: temporomandibular disorder; MG: migraine; FM: fibromyalgia.

### 3.5 Correlation between the number of comorbidities and CPM effect

The correlation analysis examined the relationship between the number of comorbidities and the CPM effect. In this study, we have analysed by selecting three groups: the TMD group had no comorbid pain, the TMD + MG group experienced one comorbid pain, and the TMD + MG + FM group faced two comorbid pain conditions. Using Pearson Correlation, we found a significantly negative correlation between the number of comorbidities and the CPM effect during single stimulation. The correlation coefficient (*r*) was −0.312 with *p* < 0.001. A similar negative correlation was observed during serial stimulation, where an *r* was −0.344 with *p * < 0.001, suggesting a meaningful yet mildly negative trend.

### 3.6 Pain modulation profile (PMP)

The cutoff value of 2.57 for WUR and the corresponding value for CPM were employed to classify participants into four pain modulation profiles. In the context of CPM, the threshold values were derived from TS relative change from the baseline for each stimulation. For single stimulation, CPM cut-off was 16.20% for the control group, 8.48% for the TMD group, 12.35% for the MG group, 9.50% for the TMD + MG group, and 10.73% for the TMD + MG + FM group. The serial stimulation had CPM cut-off points at 10.52% for the control group, 12.87% for the TMD group, 12.75% for the MG group, 14.43% for the TMD + MG group, and 12.77% for the TMD + MG + FM group. Consequently, the distribution of PMP classifications within each group was visually presented in Fig. 4. When analysing the distribution of PMP classification among the five groups, there was a significant difference among them during serial stimulation (χ^2^ = 28.85, *p* = 0.004), whereas such differences were not present in single stimulation (χ^2^ = 8.96, *p* = 0.760). Nevertheless, employing the Bonferroni correction in the context of pairwise comparisons within each category did not provide the capability to identify which specific categories differ from each other.

**Fig. 4.** Distribution of pain modulation profile (PMP) in single and serial stimulations across groups: controls (a), TMD Patients (b), Migraine Patients (c), TMD Patients with Comorbid Migraine (d), TMD Patients with Comorbid Migraine and Fibromyalgia (e). TMD: temporomandibular disorder; MG: migraine; FM: fibromyalgia; CPM: conditioned pain modulation; TSP: temporal summation of pain.

## 4. Discussion

This study investigated EPM in individuals with TMD, migraine, and fibromyalgia in comparison to a control group of healthy individuals, as well as the correlation between the CPM responses and the number of comorbidities. A total of 139 participants were predominantly female, right-handed, with an average age of 42.13 years and a mean BMI of 23.15. Although there were no significant differences in the characteristics of the participants, there is evidence that gender and age have an impact on EPM. Pain facilitation was more prominent in females and those of advanced age [47]. EPM is less efficient among females [48] and older individuals [49]. Efforts were made to adjust for age, but due to the predominantly female composition of the patient population, adjusting for gender was challenging. The study prioritized a larger, more diverse group of participants while maintaining control over other factors, such as the timing of the test and water temperature. Water temperature was closely monitored using a digital thermometer. The simple design of the CPM setup suggests potential for practical use in clinical settings with limited equipment, but the reliability of the results should be considered when interpreting findings.

The MDT and PPT tests on the hand showed no significant differences between the groups, but the face told a different story. Migraine patients were most sensitive to mechanical stimuli in the masseter muscle compared with other groups, except for TMD + MG + FM. Furthermore, the MG group consistently reported the lowest PPT on the face, with differences evident when comparing the control group. These findings contradict a prior study comparing headache with other pain conditions (TMD, FM, lower back pain, and irritable bowel syndrome) and reported the greatest degree of increased pain sensitivity in TMD and FM [50]. However, the headache population under the aforementioned study extended beyond migraineurs. Despite our discrepant findings, the rationale underlying the greatest increased sensitivity to pain in migraine compared with other pain conditions remains elusive [51], and we regrettably lacked a definitive explanation for this phenomenon. However, as the number of pain comorbidities increased, as in the TMD + MG and TMD + MG + FM groups, there was a discernible pattern of lower MDT and PPT. This observation suggests that the combination of these conditions may have a synergistic effect on pain sensitivity, a phenomenon consistent with a previous study [50]. This may underscore the possible interplay between multiple pain conditions and their collective effects on pain sensitivity.

The results from the MTS revealed interesting patterns in different stimulation scenarios. The test involved a single stimulus; there were no significant differences observed among the participants. However, in serial stimulation, notable differences emerged among groups. MTS was found to be higher in individuals with pain conditions when compared with healthy participants, except for migraine patients. These MTS results were consistent with some previous studies involving TSP using pinprick stimuli in non-trigeminal areas among TMD individuals compared with control subjects. Four studies reported an increase in MTS over the hand [13, 16], trapezius [16], and leg [52], while another study found no increase in MTS over the trapezius and hand areas [53]. The presence of migraine did not seem to significantly influence MTS responses in TMD patients. However, when fibromyalgia was present, as seen in the TMD + MG + FM group, it led to TS differences when compared with the TMD, TMD + MG and MG groups. This phenomenon could be attributed to the manifestation of widespread bodily pain and central sensitization associated with the presence of fibromyalgia. Additionally, the mean WUR did not show significant differences across the five study groups. This finding is consistent with the recent study that showed no WUR difference among TMD patients, TMD patients with migraine, and patients with headaches secondary to TMD [54]. However, another study yielded contrasting results [13]. This observation lends support to the idea that temporal summation might be an inconsistent phenomenon or that the assessment methods used may not effectively capture the phenomenon of central sensitization [55].

In the evaluation of the CPM experiment, the difference in the change from the baseline in the CS pain score serves as an indicator of pain inhibition within each group. Subsequently, the alteration in the CPM magnitude was converted into a percentage CPM effect to enable a comparison of the effectiveness of EPM among groups. The higher the CPM effect, the more efficient the EPM. In general, individuals with chronic pain conditions, particularly those in the MG, TMD + MG and TMD + MG + FM groups, displayed lower CPM responses to dynamic stimulation than healthy participants. The TMD group exhibited less CPM effect only with the single stimulation. This suggests that the ability to modulate pain in response to single and repeated stimuli is less effective in MG and TMD patients with comorbid pain compared with healthy participants. On the other hand, TMD-only patients showed a lower CPM effect compared with pain-free subjects with only single stimulation. When considering the threshold for efficient CPM, defined as the ability to inhibit at least 29% of pain [42], healthy participants had the highest proportion of efficient CPM responses. In contrast, the TMD + MG + FM groups had the lowest proportion of effective CPM responses. Altogether, our results showed that EPM was impaired in TMD patients with comorbid pain conditions and the combination of pain conditions shows a trend to reduce the efficacy of CPM.

To date, few reports have found an effect of CPM in relation to the combination of pain conditions and multiple studies have provided results on the individual pain condition in relation to EPM and CPM response. In the TMD population, the evidence regarding impaired EPM is somewhat mixed. Three studies reported impaired EPM, specifically in the trigeminal region but not in non-trigeminal regions [13, 18, 56]. Conversely, another study presented contrasting results, finding impaired EPM in both extra-segmental and intra-segmental areas in TMD patients [57]. Additionally, in one study, no EPM effect was observed in any of these sites [58]. Furthermore, when examining only the outside trigeminal area, three studies were conducted. Among these, an impairment of EPM was found in TMD patients when compared with a pain-free population in two studies [15, 59], while in one study, no such difference was found [10]. Most of these studies have provided evidence supporting intact EPM in TMD patients in non-trigeminal areas. In other words, CPM between TMD patients and healthy individuals was not different. Our results in the TMD-only group align with these findings. However, when concurrent pain conditions are present in the TMD population, it may impact their ability to engage in EPM, resulting in reduced CPM compared with healthy individuals, as demonstrated by our study results. The lack of significant CPM differences among TMD patients is consistent with a previous study that examined CPM in TMD patients with migraine and headache attributed to TMD but not in controls [54]. Furthermore, the analysis using Pearson’s correlation revealed a negative correlation between the number of comorbidities and the efficiency of CPM, for both single and serial stimulations. This implies that as the number of comorbid pain conditions increases, the ability of individuals to effectively inhibit pain decreases. Therefore, the ability to modulate pain might be an important factor to consider when managing TMD patients with co-existing pain disorders.

The distribution of PMP showed a significant difference among the five groups for serial stimulation but not for single stimulation. Interestingly, in both serial and single stimulation, the percentage of participants with either increased TSP or impaired EPM (PMP I, II, III) were higher than that in TMD-only patients and control subjects. However, applying the Bonferroni correction for pairwise comparisons within each category made it challenging to pinpoint specific group differences. A limited number of studies have reported the use of the PMP classification in chronic pain patients [13, 45] and found that a small percentage of patients had no impaired EPM and no difference in PMP classification between TMD patients and pain-free subjects [13]. This scarcity of usage may stem from its perceived lack of refinement in effectively distinguishing pain patients based on their EPM abilities. However, this study suggests that the PMP categorization approach may be valuable, especially in dynamic stimulation paradigms that showed significant pain response differences between chronic pain patients and healthy controls. This underscores the promising applicability of the PMP classification and suggests further opportunities for the development of predictive models of pain onset and personalised pain treatment strategies based on the PMP classification [60].

In terms of the different effects of stimulus modality, previous literature did not extensively explore differences in MTS and CPM outcomes between single (static) stimuli and serial (dynamic) stimuli. Our findings suggested that the cumulative effect of repeated painful stimuli, as seen in the serial test, might have a more pronounced impact on pain modulation. The result was similar to a study comparing different stimulus modalities for MTS and CPM, including heat and pressure, as well as single and serial stimuli. This prior study suggested that CPM was significantly more influenced by serial stimuli in MTS than by single stimuli in MTS [61]. However, it is crucial to acknowledge that CPM responses as well are highly dependent on the experimental design and can be influenced by considerations of its reliability [41]. The use of distinct stimuli or even variations in the intensity of the same stimulus can potentially impact the results of CPM assessments [62].

The discovery of reduced efficacy of CPM in patients suffering from various pain syndromes requires an explanation. This situation theoretically raises a dilemma resembling a “chicken and egg” scenario [60]. It suggests that either the patients originally had normal CPM efficiency, but their pain inhibition capacity had been depleted due to the persistent chronic pain, rendering them incapable of effectively reducing pain within the CPM protocol setting, or the patients initially had a less efficient CPM. CPM plays an important role in both pain research and clinical practice, enabling the assessment of a patient’s pain modulation system, predicting treatment outcomes, and guiding tailored pain management [60]. However, challenges, such as individual variability and lack of standardised protocols, need to be addressed to maximise its clinical utility in the treatment of chronic pain.

This study has several limitations to be acknowledged. Firstly, the CPM protocol used lacks standardisation for specific conditions, so it was adapted for TMD based on tailored evidence. Further research on CPM validation and variability is needed. Secondly, precise control of water temperature was critical. Unfortunately, due to limited access to equipment, we had to use an alternative insulated cooling container with a digital thermometer. These adaptations were tested and validated prior to the study. Thirdly, the population of TMD patients with comorbid fibromyalgia and migraine was small due to low prevalence and time constraints. Moreover, medications beyond NSAIDs, paracetamol, or psychiatric treatments may not have been fully excluded. While these agents could potentially influence pain modulation, their impact is likely less immediate compared with short half-life analgesics, and was therefore not specifically controlled for in the study design. Lastly, we did not include a TMD + FM group for comparison, preferring the TMD + MG + FM group to assess the impact of comorbidities within the study’s timeframe. Hence, increasing the sample size for the TMD + MG + FM group and incorporating the TMD + FM group might have potentially modified the results, rendering them more significant.

## 5. Conclusions

The coexistence of comorbid pain conditions, such as migraine and fibromyalgia, in TMD patients is correlated with a reduced capacity for endogenous pain modulation. This suggests that TMD patients struggling with concurrent pain disorders may require a more thorough assessment of their pain modulation systems. Targeted treatment strategies that systematically address these interrelated pain conditions might be crucial in improving pain management overall. This highlights the importance of a comprehensive treatment approach for individuals with multiple pain-related comorbidities.
